# Erythrocyte oxidative stress and thrombosis

**DOI:** 10.1017/erm.2022.25

**Published:** 2022-08-26

**Authors:** Alessandra Bettiol, Silvia Galora, Flavia Rita Argento, Eleonora Fini, Giacomo Emmi, Irene Mattioli, Giacomo Bagni, Claudia Fiorillo, Matteo Becatti

**Affiliations:** 1 Department of Experimental and Clinical Medicine, University of Firenze, Firenze, Italy; 2 Department of Experimental and Clinical Biomedical Sciences “Mario Serio”, University of Firenze, Firenze, Italy

**Keywords:** Erythrocyte, oxidative damage, oxidative stress, reactive oxygen species, redox regulation, thrombosis, venous thrombosis

## Abstract

Thrombosis is a common disorder with a relevant burden of morbidity and mortality worldwide, particularly among elderly patients. Growing evidence demonstrated a direct role of oxidative stress in thrombosis, with various cell types contributing to this process. Among them, erythrocytes produce high quantities of intracellular reactive oxygen species (ROS) by NADPH oxidase activation and haemoglobin autoxidation. Concomitantly, extracellular ROS released by other cells in the blood flow can be uptaken and accumulate within erythrocytes. This oxidative milieu can alter erythrocyte membrane structure, leading to an impaired erythrocyte function, and promoting erythrocytes lysis, binding to endothelial cells, activation of platelet and of coagulation factors, phosphatidylserine exposure and release of microvesicles. Moreover, these abnormal erythrocytes are able to adhere to the vessel wall, contributing to thrombin generation within the thrombus. This process results in accelerated haemolysis and in a hypercoagulable state, in which structurally impaired erythrocytes contribute to increase thrombus size, to reduce its permeability and susceptibility to lysis. However, the wide plethora of mechanisms by which oxidised erythrocytes contribute to thrombosis is not completely elucidated. This review discusses the main biochemical aspects linking erythrocytes, oxidative stress and thrombosis, addressing their potential implication for clinical and therapeutic management.

## Introduction

Thromboembolic events account for around one quarter of deaths worldwide, being the most frequent condition underlying myocardial infarction and ischaemic stroke. The incidence of thrombosis increases with age and its complications are among the major causes of long-term morbidity and poor quality of life, particularly in western countries (Ref. 1). Understanding the pathogenetic mechanisms of thrombosis is a major challenge to set up appropriate prophylactic interventions.

In recent years, many studies have focused on the role of oxidative stress, that is, a condition in which a massive reactive oxygen species (ROS) production overwhelms antioxidant defences, in inducing thrombosis (Refs 2–6). It is known that ROS can stimulate coagulation by increasing the expression of tissue factor in endothelial cells, monocytes and vascular smooth muscle cells, by directly interfering with platelet activation, as well as by inducing oxidative structural and functional modifications to key proteins involved in the coagulation cascade (including tissue factor pathway inhibitor, TFPI, protein C, thrombomodulin, fibrinogen, antithrombin). Moreover, ROS can mediate thrombo-inflammation, also via leucocyte (particularly neutrophil) hyperactivation and extracellular traps release (Ref. 7). Interestingly, while erythrocytes have traditionally been considered as playing a bystander role in haemostasis and thrombosis (Ref. 8), growing evidence suggests a direct involvement of these cells in ROS-induced thrombogenesis (Ref. 9).

Erythrocytes produce high amounts of intracellular ROS by NADPH oxidase activation and haemoglobin autoxidation. Moreover, erythrocytes can uptake extracellular ROS released by other cells in the blood flow. Accumulated ROS can induce structural changes to cell membrane, resulting in an impaired erythrocyte function and in the generation of a hypercoagulable milieu.

**Fig. 1.**
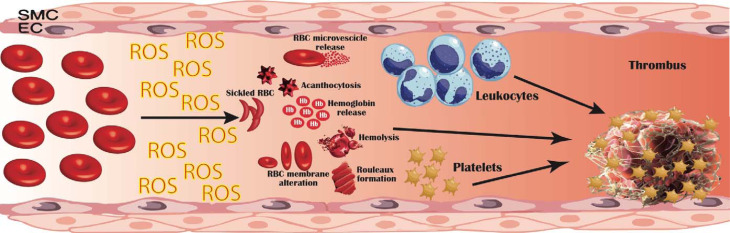
Pathogenetic mechanisms linking erythrocyte oxidative modifications to thrombosis. Erythrocytes produce high quantities of intracellular reactive oxygen species (ROS), mostly by NADPH oxidase activation and haemoglobin autoxidation; furthermore, extracellular ROS released by other cells in the blood flow can be uptaken and accumulate within erythrocytes. This oxidative milieu can alter erythrocyte membrane, leading to an impaired erythrocyte function and promoting erythrocytes lysis, binding to endothelial cells (EC), activation of platelet, coagulation factors and leucocytes. Moreover, structurally altered erythrocytes are able to adhere to the vessel wall, contributing to thrombin generation within thrombus. This process results in an accelerated haemolysis and in a hypercoagulable state, in which structurally impaired erythrocytes contribute to increase thrombus size and to reduce its permeability and susceptibility to lysis. EC, endothelial cells; RBC, red blood cells; ROS, reactive oxygen species; SMC, smooth muscle cells.

In this review, we aim to connect the dots linking erythrocytes, oxidative stress and thrombosis (Fig. 1), addressing their potential implication for the clinical and therapeutic management of thrombosis.

## Erythrocytes as leading actors in thrombosis

### Haematocrit and thrombosis

The concept that erythrocytes contribute to haemostasis was formulated more than a hundred years ago, based on the evidence that bleeding time in anaemic patients was prolonged also in the presence of a normal platelet count (Ref. 10), and that a negative correlation existed between haematocrit and bleeding time (Ref. 11). On the other hand, an abnormally high haematocrit, as observed in patients with polycythaemia vera or taking erythropoietin, has been associated with an increased risk of thrombosis (Ref. 12). Erythrocytes primarily influence blood viscosity, which increases in a nonlinear manner with haematocrit. Increased viscosity decelerates blood flow and is a component of the Virchow's triad leading to a prothrombotic state (Ref. 13). Indeed, haematocrit-related blood viscosity influences the interaction between platelets and blood vessel surfaces, with a remarkable rheological effect. Indeed, erythrocytes generally move down the centre of blood vessels, while platelets occupy marginal positions, to easily adhere at sites of vessel-wall injury (Ref. 14). In the presence of abnormally high haematocrit, platelets tend to accumulate near the vessel wall with arterial shear rates, increasing their interactions with the activated endothelium (Ref. 15). In vessels of small calibre, erythrocytes may aggregate and concentrate along the flow axis, thus further resulting in platelet margination. Moreover, as erythrocytes have a lower viscosity compared to platelets (Ref. 16), an increased haematocrit determines a reduced local viscosity (Ref. 17), which results in a decreased wall shear stress and a lower local nitric oxide (NO) release (Ref. 18). As NO prevents the activation of endothelial cells and platelets, this leads to cellular activation in a pro-thrombotic sense.

Also, at low shear rates, the peculiar erythrocyte morphology allows electrostatic interactions and cell aggregation into piled-up ‘rouleaux’ structures, which cause an increased viscosity and hydrodynamic resistance (Ref. 19). This phenomenon is more common in larger venous vessels at lower shear rates, such as in the lower limbs, which indeed are an elective site of venous thrombosis (Ref. 20). Notably, fibrinogen is essential for the formation of rouleaux under low shear conditions (Ref. 21) as it is able to bridge nearby cells, stimulating aggregates formation; the connection between fibrinogen and erythrocytes seems to be mediated by an integrin receptor on erythrocytes membrane, the *β*3 integrin (Ref. 22) and/or the integrin-associated protein (CD47) (Ref. 23).

### Erythrocyte structure and thrombosis

Even when haematocrit is within physiological ranges, erythrocytes can promote a pro-thrombotic state following structural and functional cell alterations. Erythrocytes are uniquely deformable cells with a characteristic biconcave shape capable of undergoing reversible shape changes into a bullet-like shape each time they pass inside microvessels. This morphology is essential to guarantee oxygen/carbon dioxide exchange between tissues and blood; indeed, by maximising the active contact area between erythrocytes and the vessel wall, as a result of erythrocyte deformation and high surface-to-volume ratio, gas exchange is optimised (Ref. 24).

In some diseases, including sickle cell disease, *β*-thalassemia, haemolytic anaemias and hereditary stomatocytosis, as well as in chronic conditions such as diabetes, hypertension and coronary heart disease, erythrocytes show more rigid and less deformable structure (Refs 25, 26). This results in a lower ability to squeeze through capillaries and in an increased platelet margination, contributing to a prothrombotic state (Refs 27, 28).

Also, in sickle cell disease and *β*-thalassemia, the damaged erythrocyte membrane externalises phosphatidylserine, a negatively charged phospholipid which is physiologically located on the cytoplasmic side of the membrane. Phosphatidylserine exposure provides an active surface for prothrombin activation, determining a high thrombotic potential (Ref. 29).

When exposed to high shear rates, inflammation, or in the above-mentioned diseases, erythrocytes can also generate microscopic extracellular membranous structures named microvesicles or microparticles, as a result of apoptosis activation or aging (Ref. 30).

Microparticles enhance thrombin generation via the expression of phosphatidylserine and tissue factor, via the internalisation of free haeme and its transfer to vascular endothelium, as well as via the amplification of systemic inflammation through thrombin-dependent complement activation (Ref. 31).

### Erythrocytes and clot structure

Erythrocytes not only influence clot formation but also clot structure. Growing evidence shows that erythrocytes may be integrated into the thrombus, through unique liaisons with activated endothelial cells and/or exhibited subendothelial matrix (Ref. 32). Under normal circumstances, mature erythrocytes are not able to interface with endothelium; conversely, structurally and functionally altered erythrocytes (as observed in sickle cell disease, malaria or diabetes) show an increased stickiness and adhesion to the vascular endothelium, contributing to microvascular occlusions associated with thrombosis (Ref. 33). Incorporation of erythrocytes in the thrombus influences fibrin network by increasing fibre diameter thus impacting on the viscoelastic clot properties (Ref. 34). In contracted clots and thrombi, erythrocytes have been shown to undergo a shape transformation from their native biconcave shape to a close-packed polyhedral structures covered by platelets and fibrin (polyhedrocytes) (Ref. 35). Polyhedrocytes have been reported in coronary arterial thrombi from patients after myocardial infarction (Ref. 35) and in pulmonary embolia (Ref. 36). This structure decreases clot permeability to fibrinolytic agents, thereby increasing its resistance to lysis.

Interestingly, it has been suggested that erythrocytes can display also antithrombotic properties. In particular, haemoglobin deoxygenation is followed by an allosteric transition stimulating NO release from cysteine *β*93 of haemoglobin, with consequent capillary and postcapillary venules dilatation and inhibition of platelet reactivity (Ref. 37). Moreover, ATP released from erythrocytes at low pH/reduced PO_2_ conditions or shear stress, can stimulate the activation of endothelial cell purinergic receptors, increasing NO production (Ref. 38). Also, it has been demonstrated that erythrocyte expression of ectoenzyme degrading ADP to AMP exerts antithrombotic properties by suppressing platelet aggregation (Ref. 39).

Therefore, erythrocytes structural and functional integrity displays critical roles in physiological haemostasis and thrombosis.

#### Erythrocyte and platelet interactions

Erythrocytes interact with platelets via different mechanisms. As previously described, erythrocytes exert a rheological effect, concentrating along the flow axis and causing platelet margination (Refs 16, 17). As a consequence, platelets are in close contact with the vessel wall, where they can interact with other clotting factors.

Moreover, as erythrocytes have a lower viscosity compared to platelets (Ref. 16), an increased haematocrit determines a reduced local viscosity (Fahraeus effect), except in capillaries that are smaller than erythrocytes, where the viscosity increases because of the presence of platelets (Ref. 17).

The reduced viscosity near the vessel wall determines a decreased wall shear stress and a reduced NO release (Ref. 18), leading to cellular activation in a pro-thrombotic sense.

Erythrocytes can interact directly with platelets at venous shear rates, although erythrocyte-platelet binding has been described also in the so-called ‘white’ arterial thrombi mainly composed of activated platelets and fibrin (Ref. 40).

Beside straight adhesive interactions (Refs 41, 42), erythrocytes can stimulate platelet degranulation and aggregation via chemical signalling, (i.e. through the release of ATP and ADP under low pO2 and low pH), as well as through the action of extracellular haemoglobin released from damaged erythrocytes (Ref. 43). Indeed, haemoglobin is a strong NO scavenger, and the release of extracellular haemoglobin from damaged erythrocytes determines a reduction in NO bioavailability, thus preventing the suppressive effect of NO on platelet activation (Ref. 43). Concomitantly, the release of arginase from damaged erythrocytes determines the cleavage of L-arginine, a substrate for NO production (Ref. 43).

## Oxidative stress and thrombosis

In the last years, a prominent role of oxidative stress in regulating both endothelial dysfunction and thrombus formation is emerging.

The importance of oxidative stress in thrombogenesis was first demonstrated in an experimental mice model of thrombosis (mice lacking functional eNOS), where NO deficiency was significantly associated to arterial thrombosis. These mice showed lower bleeding times if compared to wild-type animals (Ref. 44). Later on, it has been shown that a moderate iron overburden significantly stimulates thrombus formation, via a defective vasoreactivity as well as via an enhanced ROS production (Ref. 45).

### ROS interfere with pro- and anticoagulant molecules

ROS can interfere with the coagulation process via a plethora of multiple, interconnected mechanisms. ROS, mostly generated by NOX enzymes, can directly stimulate the coagulation cascade by increasing the expression of tissue factor in endothelial cells, monocytes and vascular smooth muscle cells (Refs 46–48). ROS can also promote a procoagulant state via oxidative modification of proteins involved in the coagulation pathway, such as the anticoagulant proteins protein C (Ref. 49), thrombomodulin (Ref. 50) and the TFPI, resulting in their inactivation (Ref. 51). Indeed, in mice models lacking superoxide dismutase (*SOD-/-* mice), larger, rapidly growing venous thrombi were observed, due to an impaired SOD1-mediated protein C activation (Ref. 52). Also, the heparin-binding capacity of antithrombin is decreased following oxidation by hydrogen peroxide (Ref. 53) or lipid peroxide (Ref. 54).

Furthermore, lipid oxidation can inactivate the anticoagulant function of protein Z-dependent protease inhibitor, a specific inhibitor of membrane-associated factor Xa (FXa) (Ref. 55).

Similarly, it has been observed that leucocyte-produced ROS can oxidise fibrinogen, altering its secondary structure and the overall clot architecture, characterised by reduced porosity and by tight fibrin network with filaments of decreased average size. Also, these oxidative alterations result in an impaired fibrinogen function, both in terms of thrombin-catalysed fibrin polymerisation and fibrin susceptibility to plasmin-induced lysis. This mechanism has been linked to increased thrombosis risk in Behcet's syndrome (Refs 4, 56), cirrhosis (Ref. 5), and it has been also described in post-acute myocardial infarction (Ref. 6), pulmonary hypertension (Ref. 57) and pulmonary embolism (Refs 58, 59).

### ROS and platelets

Besides affecting the activity of pro- or anti-coagulant molecules through oxidative modification, ROS can also directly interfere with platelets and other cells involved in haemostasis and thrombosis.

Intraplatelet ROS can activate platelets, by oxidising arachidonic acid, generating isoprostanes (Ref. 60); this mechanism has been linked with an increased risk of deep venous thrombosis in patients with hypercholesterolaemia (Ref. 61), diabetes mellitus (Ref. 62), homozygous homocystinuria (Ref. 63) and in obese women (Ref. 64). Concomitantly, ROS can also indirectly promote platelet activation by negatively regulating mechanisms of platelet inhibition, such as NO scavenging (Ref. 65). In hyperhomocysteinemia superoxide formation by hyperactive platelets has been described as one of the key pathways contributing to arterial thrombosis in this condition (Ref. 66).

### ROS and leucocytes

ROS also modulate platelet-leucocyte interactions: ROS produced by NOX2 can affect the expression of P-selectin (CD62) and CD40L, that are transferred to the platelet surface upon activation. P-selectin and CD40L promote leucocyte recruitment and activation (Refs 67, 68) and their levels are associated with an increased risk of venous thromboembolism in various conditions (Ref. 69), such as in Behçet's syndrome (Refs 67, 68). Concomitantly, ROS can induce leucocyte recruitment via different complementary mechanisms: they can directly act as a chemoattractant for neutrophils and monocytes, mostly via upregulation of IL-8 (Ref. 70) and of monocyte chemotactic protein-1 (MCP-1) production, respectively (Ref. 71). Moreover, they can increase the expression of leucocyte adhesion molecule expression (such as platelet-endothelial cell adhesion molecules-1, PECAM-1) and promote leucocyte endothelial adhesion (Ref. 72).

Also, ROS can activate mast cells, which on their turn produce ROS, mostly via NOX2, with consequent redox-sensitive calcium channels activation, increase in cytoplasmic calcium concentrations required for the induction of mast cell degranulation (Ref. 73) and leucocyte recruitment and activation (Ref. 74). The leucocyte-ROS axis is particularly relevant in the process of thrombo-inflammation, which sustains thrombotic events in various immune-mediated conditions such as thrombosis in Behçet's syndrome (Ref. 4).

In Behçet's syndrome, ROS have been shown to stimulate neutrophils to release extracellular traps (NETs) (Ref. 7). NETs are structures composed of cell-free DNA, histones, microbicidal proteins and proteases, that are extruded by dead neutrophils, mostly by low-density granulocytes (LDGs), following infective or inflammatory stimuli (Ref. 75). NETs can directly induce thrombogenesis (Ref. 76), by activating the intrinsic and extrinsic coagulation pathways, and by enhancing thrombin production in plasma, probably via histone/polyphosphate triggering (Ref. 76). Concomitantly, NETs can stimulate neutrophils to further produce ROS, in a self-sustaining process.

Also, in Behçet's syndrome, leucocyte ROS levels have been correlated with a peculiar profile of circulating miRNAs (i.e. small non-coding RNAs that act as post-transcriptional regulators of gene expression) affecting pathways related to cell-matrix interaction, oxidative stress and blood coagulation (Refs 77, 78), suggesting a contribution of epigenetic mechanisms in ROS-induced thrombo-inflammation.

## Connecting the dots: the erythrocyte-ROS axis in thrombosis

As described in the previous paragraphs, erythrocyte can contribute to thrombogenesis via different mechanisms and growing studies suggest a key role of oxidative stress in linking erythrocytes to thrombosis (Supplementary Table 1).

Erythrocytes have a plethora of enzymatic (e.g. superoxide dismutase, catalase, glutathione peroxidase and peroxiredoxin-2 (PRDX-2)) and non-enzymatic antioxidant defences. Among the latter, reduced glutathione (GSH) is a ubiquitous intracellular antioxidant which inhibits free radical formation and more generally acts as a redox buffer, detoxifier and chemokine scavenger. Erythrocytes can export GSH at a constant rate of ~ 21 nmol/h/ml erythrocytes, contributing to the extracellular GSH reservoir (Ref. 79). GSH is synthesised *de novo* from cysteine, glycine and glutamate by the enzymes, *γ*-L-glutamate L-cysteine ligase and glutathione synthetase (Ref. 79). Reduced GSH concentration has been reported in various conditions characterised by an increased cardiovascular risk, such as diabetes mellitus (Ref. 80), hypertension (Ref. 81), haemodialysis and peritoneal dialysis (Ref. 82), and is considered as an indicator of an impaired oxidative stress.

Within erythrocytes, oxidative stress can be sustained by ROS released from neutrophils and macrophages into the plasma and taken up by erythrocytes, particularly in microcirculation, where the erythrocytes are in close contact with the vasculature (Ref. 83). Also, erythrocyte also contains NADPH oxidases, which can generate endogenous ROS (Ref. 84). Endogenous and exogenous ROS induce oxidation of iron contained in haemoglobin, from Fe^2+^ containing haemoglobin to Fe^3+^-containing methaemoglobin.

Fe^3+^ induces iron-dependent free radical generation (Fenton reaction) which causes lipid peroxidation, haemolysis and endothelial perturbation. This triggers a haemolysis/oxidative cycle, which promotes vascular injury, thrombus formation and atherothrombotic events (Ref. 85) as observed in severe haemolytic syndromes (Ref. 85).

The oxidised Fe^3+^ methaemoglobin can be converted back into the reduced form by a cytochrome b5 reductase. However, if the reducing equivalents for this enzyme are scarce, haeme is further degraded to quaternary compounds with consequent ROS formation (Ref. 86).

ROS damage erythrocyte membrane (Ref. 87), reduce cell deformability and induce cell lysis, by triggering a molecular signalling cascade with the activation of Ca^2+^ permeable cation channel (Ref. 88). The influx of Ca^2+^ activates Ca^2+^-sensitive K^+^ channels, leading to phosphatidylserine exposure on the erythrocyte membrane (Ref. 88). This provides an active surface for prothrombin activation: it has been postulated that even a small fraction of erythrocytes exposing phosphatidylserine can lead to thrombin generation, accounting for up to 30–40% of the thrombin-generating potential of whole blood (Ref. 89). Notably, in a mouse model of sickle cell disease, reducing erythrocyte ROS production with manganese porphyrins, which suppress erythrocyte NOX activity (Ref. 90) was found to result in a reduced phosphatidylserine exposure and improved eryptosis (Ref. 91).

Beside directly stimulating thrombin generation, phosphatidylserine exposure on the erythrocyte membrane stimulates the release of microvesicles (Ref. 92) with a high thrombotic potential (Ref. 29), as previously described and considered a promising target for the treatment of thrombotic disorders (Ref. 93). Oxidation-induced damage on erythrocyte membrane further induces haemolysis. Under physiological conditions, the release of free haemoglobin and haeme can be inactivated by plasma haptoglobin and hemopexin (Refs 94, 95) leading to their phagocytosis (Ref. 96). Conversely, oxidised haemoglobin has a low affinity for haptoglobin, resulting in an impaired plasma clearance and in an increased release of haeme and iron (Ref. 97). Free redox-active haeme translocate into endothelial cells, triggering H_2_O_2_-mediated endothelial damage and overwhelming intracellular antioxidant defences.

Moreover, extracellular haeme derived from lysed erythrocytes mediates additional pro-thrombotic mechanisms: it stimulates neutrophil recruitment and NETosis (Ref. 98), as observed in sicke cell disease (Ref. 98) and promotes NLRP3 inflammasome activation and cytokine and lipid mediator production in macrophages (Ref. 99) which have been shown to potentiate venous thrombosis (Ref. 100). Specifically, free haemoglobin and haeme can stimulate the nuclear factor *κ*B (NF-*κ*B) under the control of a Toll-like receptor (TLR)-signalling pathway (Refs 101, 102) leading to the activation of hypoxia-inducible factor (HIF)-1*α* and HIF-2*α* (Ref. 103) which further induce inflammation, vasoconstriction and increase endothelial permeability (Ref. 103).

Furthermore, free haemoglobin can upregulate the expression of functional tissue factors in macrophages and desensitises tissue factor to the effects of antioxidants, such as glutathione or serum (Ref. 104). Also, it can scavenge NO, thereby impairing its regulatory effects on vasocostriction, endothelial adhesion molecule expression and platelet activation and aggregation, in a pro-thrombotic sense (Ref. 105). Free haeme can induce platelet activation also by binding to glycoprotein-1b alpha (GPIb*α*) on platelets (Ref. 106), as well as through C-type lectin-like receptor-2 (CLEC-2) (Ref. 107).

A direct role of erythrocyte oxidative stress has been described in retinal vein occlusion, a condition characterised by vision loss resulting from hypoperfusion and hypoxia of the retina. Increased erythrocyte oxidative stress levels were found in patients with retinal vein occlusion; also, erythrocyte-derived ROS and erythrocyte lipid peroxidation were found to positively correlate with erythrocyte membrane viscosity and deformability (Ref. 108).

Similarly, in patients with cochlear vascular occlusion leading to sudden sensorineural hearing loss, a significant structural and functional involvement of erythrocyte membrane alterations was found, associated with enhanced levels of membrane lipid peroxidation and intracellular ROS production. Notably, in vitro experiments demonstrated that ROS display a critical role in impairing erythrocyte membrane fluidity (Ref. 109).

Of major note, ROS-induced erythrocyte modifications are particularly relevant during aging. An age-dependent increase in erythrocyte oxidative stress markers paralleled by an age-dependent decline in the total plasma antioxidant capacity has been reported (Refs 110–112). In rat models, an increase in plasma membrane redox system activity, lipid peroxidation and erythrocyte malondialdehyde has been reported in senescent erythrocytes, paired by a reduced L-cysteine influx and a consequent decrease in intracellular GSH (Ref. 113).

Beside erythrocytes, also platelets exhibit a progressive impairment in redox status during aging, with a marked increase in oxidative stress, hyperactivation and apoptotic markers, although this trend is reverted in old subjects (80–100 years) (Ref. 114). Accordingly, erythrocyte and platelet oxidative stress has been suggested as one of the major mechanisms sustaining the pathogenesis of thrombotic events during aging, with potentially relevant implications in terms of thrombotic prophylaxis and treatment (Refs 115, 116). In aging rat models, rapamycin, particularly when combined with metformin, was found to be a promising age-delaying agent, able to restore altered levels of redox biomarkers in erythrocytes (Refs 117, 118).

## Therapeutic implications

Understanding the role of the erythrocyte-oxidative stress axis in inducing thrombosis offers the possibility of setting up new prophylactic strategies for cardiovascular preventions (Table 1).

**Table 1.** Therapeutic implications

Therapeutic interventions	Main protective action against ROS-induced thrombosis
Pharmacological therapies	
Antihypertensive agents: Angiotensin-converting enzyme (ACE) inhibitors	Block the conversion of Ang I to Ang II, which induces endothelial dysfunction via promoting leucocytes recruitment, ROS production, LDL oxidation and NO degradation
Lipid-lowering agents: Statins	Block Rho and Rac activation (a major source of ROS production in vasculature), thus reducing endothelial activation, while increasing the expression of eNOS and the endothelial production of the vasorelaxant NO. Contribute to resolution of venous thrombi.
Antiplatelets (aspirin) and anticoagulants (Xa inhibitor, rivaroxaban)	Reduce NOX2-mediated platelet ROS production
Thioredoxin inhibitors	Attenuate platelet function and thrombus formation
Antidiabetic drugs (alogliptin)	Suppress stress-induced free fatty acid release, oxidative stress, adipose tissue inflammation and prothrombotic state in a dose-dependent manner, and improve insulin sensitivity
***Non-pharmacological interventions***	
Vitamins	
Vitamin E (tocopherols and tocotrienols)	Pleiotropic antithrombotic effects, reduces the expression and release of endothelial adhesion molecules, prevents leucocyte/endothelial cell interactions, inhibits smooth muscle cells proliferation, inhibits the formation of platelet-leucocytes aggregates. Lipoperoxyl radical-scavenging actions.
Vitamin C	Enhances endothelium-dependent vasodilation by increasing NO availability.
Dietary regimens	
Mediterranean diet	Downregulates sNOX2-dp (soluble NOX2-derived peptide) and F2-isoprostanes
Antioxidants (beer, red wine, olive oil)	Decrease ROS accumulation and inhibit platelet activation
Nattokinase (traditional Japanese food Natto)	Inhibits LPS-mediated TLR-4 and NOX2 signalling in macrophages
Butyrate	In Behçet syndrome, reduces ROS production and ROS-mediated fibrinogen structural and functional alternations

Ang, angiotensin; LDL, low-density lipoprotein; LPS, Lipopolysaccharide; NO, nitric oxide; NOX, NADPH oxidase; ROS, reactive oxygen specifies; TLR, toll-like receptor.

### Pharmacological therapies

Angiotensin-converting enzyme (ACE) inhibitors are among guideline-recommended first-line therapies in patients with hypertension to reduce the related risk of atherosclerotic disease and cardiovascular events. Growing evidence suggests that these agents exert cardiovascular effects that go beyond blood pressure reduction (Refs 119–121).

ACE inhibitors block the conversion of Ang I to Ang II, which induces endothelial dysfunction via promoting leucocytes recruitment and ROS production, with consequent enhanced LDL oxidation and NO degradation (Ref. 120).

Similarly, statins are lipid-lowering agents recommended in patients with hypercholesterolemia. In vitro and in vivo studies showed that statins can modulate the atherosclerotic process, through mechanisms additive to blood cholesterol reduction, that include anti-inflammatory and antioxidant actions (Refs 122–124).

Indeed, statins can interfere with leucocyte migration, proliferation and leucocyte/endothelial interactions (Ref. 125). Also, statins (particularly atorvastatin) can block Rho and Rac activation, thus reducing endothelial activation, while increasing the expression of eNOS and the endothelial production of the vasorelaxant NO. As the activation of Rho family members is a major source of ROS production in the vasculature, statins can counteract oxidative stress mechanisms which contribute to an increased risk of thrombotic events (Refs 126–128). Statins were found to contribute also to the resolution of venous thrombi, although the mechanism has not fully clarified (Ref. 129).

Similar effects have been reported for antiplatelets (aspirin), anticoagulants (Xa inhibitor, rivaroxaban), thioredoxin inhibitors (Refs 130–132) and the oral anti-diabetic drug alogliptin (Ref. 133).

### Vitamins

Among non-pharmacological agents, vitamins, particularly A, C and E, are known to reduce the risk of atherosclerosis and related complications.

In vitro and in vivo studies report that vitamin E exerts pleiotropic antithrombotic effects by reducing the expression and release of endothelial adhesion molecules, preventing leucocyte/endothelial cell interactions. Also, it counteracts cholesterol-induced atherosclerotic lesions progression by inhibiting smooth muscle cells proliferation and it can inhibit the formation of platelet-leucocytes aggregates and the activation of the clotting system (Refs 134–136). Notably, natural vitamin E consists of a family of eight compounds, four tocopherols and four tocotrienols. All tocopherols and tocotrienols are potent antioxidants with lipoperoxyl radical-scavenging actions able to counteract oxidative stress. In patients with type 2 diabetes mellitus and the haptoglobin 2-2 genotype presenting increased oxidative stress levels, vitamin E was found to reduce the risk of cardiovascular events (Ref. 137); however, the cardioprotective effect of vitamin E supplementation in the general population as well as in other high-risk setting was disappointing (Refs 138–140).

Vitamin C was found to enhance endothelium-dependent vasodilation, both in normotensive and hypertensive subjects (Ref. 141), thanks to its effects on NO availability (Ref. 142). However, contrasting findings were reported on the benefits of vitamin C supplementation for cardiovascular prevention.

In another study, it was shown that vitamin C (0.5–5 mM) increased the procoagulant activity of freshly isolated human erythrocytes, particularly those from cancer patients, via the externalisation of phosphatidylserine and the formation of phosphatidylserine -bearing microvesicles. Also, in rat models, the intravenous injection of vitamin C (0.5–1.0 g/kg) significantly increased thrombosis. (Ref. 143).

### Dietary regimens

Diets, especially high-fat or high-carbohydrate diets, can increase oxidative stress by elevating the levels of protein carbonylation and lipid peroxidation while impairing antioxidant defences (Ref. 144). In obese patients, insulin resistance greatly increases oxidative stress, thus contributing to the increased risk of hypertension, dyslipidaemia, type 2 diabetes, atherosclerosis and non-alcoholic fatty liver disease associated with this condition (Ref. 145).

The cardioprotective role of specific nutritional regimens has been widely investigated. In a prospective cohort study on more than seven hundred patients with atrial fibrillation, the cardioprotective role of Mediterranean diet was investigated. Results indicated that adherence to Mediterranean diet could be associated with a reduction of cardiovascular events, through an antioxidant effect, as shown by a downregulation of sNOX2-dp (soluble NOX2-derived peptide) and F2-isoprostanes during this dietary regimen (Ref. 146).

Moreover, xanthohumol contained in beer, was found to prevent arterial and venous thrombosis in mice, by decreasing ROS accumulation and inhibiting platelet activation (Ref. 147). Similar effects were suggested for antioxidants contained in red wine (Refs 148, 149) and olive oil (Ref. 150). Also, nattokinase, a serine protease from the traditional Japanese food Natto, displays anti-inflammatory and anti-oxidative stress activities by inhibiting LPS-mediated TLR-4 and NOX2 signalling in macrophages, thereby exerting a protective effect against inflammation-induced thrombosis (Ref. 151).

More recently, tailored nutritional interventions have been investigated to counteract thrombo-inflammation in peculiar chronic immune-mediated diseases, such as Behçet syndrome. Behçet syndrome displays a peculiar gut microbiota fingerprint, with an impaired production of short-chain fatty acids, especially butyrate (Ref. 152), which can exert protective effects against cardiovascular diseases (Ref. 153). Butyrate-enriched dietary interventions were recently found to reduce ROS production and ROS-mediated fibrinogen structural and functional alternations in these patients (Ref. 154) paving the way for new cardioprotective therapies in this condition.

## Concluding remarks

The erythrocyte/ROS axis is involved in the regulation of various processes that promote thrombosis. An impaired redox state induces erythrocyte membrane damage, leading to membrane fluidity alterations and decreased deformability. These changes impair erythrocyte function in the haemostatic process, promoting thrombosis via haemolysis, phosphatidylserine exposure, microvescicle release, induction of platelet activation and aggregation and vascular injury. Oxidised erythrocytes not only promote thrombus formation but also contribute to increase its size and to reduce its permeability and susceptibility to lysis and studies have suggested that the role of erythrocytes is particular once the thrombogenetic process has started and erythrocytes are entrapped within the growing thrombus (Ref. 155). However, the wide plethora of mechanisms by which oxidised erythrocytes contribute to thrombosis is not completely elucidated.

Deeping current knowledge on the mechanisms linking ROS and erythrocytes and their crosstalk with leucocytes, platelets and pro- and anti-coagulant molecules will pave the way to new therapeutic strategies for reducing thrombosis risk, particularly in conditions characterised by a sustained thrombo-inflammatory milieu.

## Supplementary material

For supplementary material accompanying this paper visit http://doi.org/10.1017/erm.2022.25. click here to view supplementary material
