# Supplementary material for: Erythrocyte oxidative stress and thrombosis
Source: Expert Rev Mol Med. 2022 Aug 26;24:e31. doi: 10.1017/erm.2022.25 (PMC9884766; doi:10.1017/erm.2022.25)
Supplement: Supplementary file 1 [file S1462399422000254sup001.docx]

**Supplementary table 1. Main literature studies supporting a direct link between erythrocyte-derived ROS and thrombosis**

| ***Study reference*** | ***Experimental/clinical setting*** | ***Main results*** |
| --- | --- | --- |
| Liu M. Erythrocyte Fraction in Thrombi Is Increased with Serum Iron by Influencing Fibrin Networks via Oxidative Stress. Oxid Med Cell Longev. 2021 (156) | 30 patients with acute ischemic stroke  + rats | Serum iron concentration is related to erythrocyte-rich thrombi and is positively associated with the erythrocyte fraction in thrombi in vivo. Inhibiting oxidative stress in vivo shows that the erythrocyte fraction in thrombi and the tightness of fibrin networks significantly increases in the iron group than those in the iron with oxidative stress inhibitor group and control group. |
| Czubak-Prowizor K. Red blood cell supernatant increases activation and agonist-induced reactivity of blood platelets. Thromb  Res. 2020 (157) | Blood samples from 17 healthy donors | Transfusion of aged packed red blood cells (PRBCs)  may result in the hyper-activity of platelets, presumably mediated by free hemoglobin and/or the products of its breakdown, accumulating in the PRBC milieu, and their ability to trigger the ROS generation. |
| Sudnitsyna J. Microvesicle Formation Induced by Oxidative Stress in Human  Erythrocytes. Antioxidants (Basel). 2020 (158) | Blood samples from healthy donors | During oxidative stress, erythrocytes eliminate hemichrome  by vesiculation in order to sacrifice the cell itself, thereby prolonging lifespan and delaying the untimely clearance of in all other respects healthy RBCs. |
| Thamilarasan M. Mn porphyrins as a novel treatment targeting sickle cell NOXs to reverse and prevent acute vaso-occlusion in vivo. Blood Adv. 2020 | Sickle mice | Mn porphyrins can suppress sickle erythrocyte NOX activity, reducing leukocyte and sickle eyrthrocyte adhesion, diminishing leukocyte rolling, restoring blood flow, and increasing survival rate. |
| MacKinney A. Disrupting the vicious cycle created by NOX activation in sickle erythrocytes exposed to hypoxia/reoxygenation prevents adhesion and vasoocclusion. Redox Biol. 2019 (90) | Blood samples from patients with sicke cell disease | Sickle erythrocyte ROS create an intracellular positive feedback loop with ERK1/2 and GRK2 to mediate sickle erythrocyte adhesion to endothelium in vitro, and vasoocclusion in a mouse model of vasoocclusion in vivo |
| Becatti M. Erythrocyte Membrane Fluidity Alterations in Sudden Sensorineural Hearing Loss Patients: The Role of Oxidative Stress.  Thromb Haemost. 2017 (109) | 35 patients with sudden sensorineural hearing loss and 35 healthy subjects | Significant structural and functional involvement of erythrocyte membrane alterations in sudden sensorineural hearing loss, as well as elevated levels of membrane lipid peroxidation and intracellular ROS production. Erythrocyte-derived ROS and erythrocyte lipid peroxidation positively correlate with whole blood viscosity and erythrocyte deformability. In vitro experiments demonstrate that ROS display a key role in erythrocyte membrane fluidity. |
| Becatti M. Erythrocyte oxidative stress is associated with cell deformability in patients with retinal vein occlusion. J Thromb Haemost. 2016 (108) | 128 patients with retinal vein occlusion | Erythrocyte-derived ROS and erythrocyte lipid peroxidation are significantly and positively correlated with erythrocyte membrane viscosity and deformability. |
| Yunoki K. Erythrocyte-rich thrombus aspirated from patients with ST-elevation myocardial infarction: association with oxidative stress and its impact on myocardial reperfusion. Eur Heart J. 2012 (159) | Aspirated thrombi from 178 STEMI patients | Erythrocyte-rich thrombi contain more inflammatory cells and reflect high thrombus burden, leading to impaired myocardial reperfusion in STEMI patients. |

*PRBC: packed red blood cells (PRBCs); ROS: reactive oxygen species; STEMI: ST-elevation myocardial infarction*
